# Neurocognitive and Clinical Predictors of Long-Term Outcome in Adolescents at Ultra-High Risk for Psychosis: A 6-Year Follow-Up

**DOI:** 10.1371/journal.pone.0093994

**Published:** 2014-04-04

**Authors:** Tim Ziermans, Sanne de Wit, Patricia Schothorst, Mirjam Sprong, Herman van Engeland, René Kahn, Sarah Durston

**Affiliations:** 1 Department of Clinical Child and Adolescent Studies, Leiden University, Leiden, the Netherlands; 2 Department of Psychiatry, University Medical Center Utrecht, Rudolf Magnus Institute of Neuroscience, Utrecht, the Netherlands; King's College London, United Kingdom

## Abstract

**Background:**

Most studies aiming to predict transition to psychosis for individuals at ultra-high risk (UHR) have focused on either neurocognitive or clinical variables and have made little effort to combine the two. Furthermore, most have focused on a dichotomous measure of transition to psychosis rather than a continuous measure of functional outcome. We aimed to investigate the relative value of neurocognitive and clinical variables for predicting both transition to psychosis and functional outcome.

**Methods:**

Forty-three UHR individuals and 47 controls completed an extensive clinical and neurocognitive assessment at baseline and participated in long-term follow-up approximately six years later. UHR adolescents who had converted to psychosis (UHR-P; *n* = 10) were compared to individuals who had not (UHR-NP; *n* = 33) and controls on clinical and neurocognitive variables. Regression analyses were performed to determine which baseline measures best predicted transition to psychosis and long-term functional outcome for UHR individuals.

**Results:**

Low IQ was the single neurocognitive parameter that discriminated UHR-P individuals from UHR-NP individuals and controls. The severity of attenuated positive symptoms was the only significant predictor of a transition to psychosis and disorganized symptoms were highly predictive of functional outcome.

**Conclusions:**

Clinical measures are currently the most important vulnerability markers for long-term outcome in adolescents at imminent risk of psychosis.

## Introduction

A major aim of twenty-first century schizophrenia research is to optimize the prediction of psychosis onset to guide initiatives on early intervention. The establishment of ultra-high risk (UHR) criteria for psychosis [1], [2] has greatly enhanced our ability to study individuals relatively close temporally to the onset of psychosis and thereby our ability to improve prediction. Although many UHR studies focus on transition to psychosis as the main outcome of interest, this arbitrary threshold is arguably a suboptimal method for identifying individuals truly at risk of poor outcome [3], [4]. Instead, it has been proposed that studies should focus more on functional outcomes, such as the level of cognitive impairment, psychosocial functioning and clinical status [5]–[8]. Surprisingly, such measures have received little attention as an outcome measure until recently, despite the general recognition that functional outcome is likely to be highly associated with long-term social and occupational functioning [9], [10].

In this study we investigated the predictive power of both neurocognitive and clinical variables in predicting both transition to psychosis and functional outcome. In addition, we focused on a group of young adolescents (18 years or younger at baseline), as it is currently unclear whether predictive accuracy of neurocognitive and clinical markers is comparable between younger and older individuals with at-risk symptoms [11]. A group of young adolescents at UHR and typically developing controls (TDC) were recruited at baseline and participated in a comprehensive neurocognitive assessment. Subsequently, individuals were followed up for a period of approximately six years to monitor clinical outcome. Our first aim was to determine whether neurocognitive variables could discriminate between TDC and UHR individuals at baseline and predict transition to psychosis, both by themselves and in combination with clinical parameters. Secondly, we investigated whether baseline cognitive functioning and clinical parameters could predict long-term functional outcome of UHR individuals. Based on recent meta-analytic evidence [12], we hypothesized (1) that neurocognitive functioning would be relatively impaired in UHR individuals compared to TDC, and (2) that for the UHR individuals, impairments in cognitive functioning would predict whether they later converted to psychosis (UHR-P) or not (UHR-NP), as well as long-term functional outcome [6], [7]. Finally, it was expected (3) that the combination of neurocognitive and clinical parameters would provide the best prediction of long-term clinical outcome [13]–[15].

## Methods

### Participants

All data were collected at the Department of Psychiatry at the University Medical Center Utrecht in The Netherlands. Participants were between 12 and 18 years of age at the time of recruitment and were included after informed consent was given. Participants and parents were provided with a comprehensive written and oral explanation of all procedures. After full disclosure of the study purpose and procedure, written consent was obtained from both the participants and their parents for individuals younger than 18 years of age. During follow-up assessments, individuals aged 18 years or older provided their own informed consent. All clinical investigation has been conducted according to the principles expressed in the Declaration of Helsinki. The study was approved by the Dutch Central Committee on Research Involving Human Subjects.

Recruitment details of the project have been described in previous publications [16], [17]. Briefly, the UHR group represented help-seeking adolescents referred by general practitioners or other psychiatric clinics. Participants had to fulfill at least one of the following criteria: 1) attenuated positive symptoms (APS), 2) brief, limited, or intermittent psychotic symptoms (BLIPS), 3) genetic risk for psychosis, combined with a deterioration in overall level of social, occupational/school, and psychological functioning in the past year (GRD) or 4) two or more of a selection of nine basic symptoms used to assess mild cognitive disturbances (COGDIS). The first three inclusion criteria were assessed using the Structured Interview for Prodromal Syndromes (SIPS) and the accompanying Scale of Prodromal Symptoms (SOPS) [18]. The fourth inclusion criterion was assessed using the Bonn Scale for the Assessment of Basic Symptoms-Prediction List (BSABS-P) [19]. Exclusion criteria consisted of a past or present psychotic episode lasting longer than one week, traumatic brain injury or any known neurological disorder, and verbal intellectual functioning (VIQ) <75. The control group consisted of TDC recruited through secondary schools in the region of Utrecht. They were excluded if they met one of the UHR-criteria, if they or any first degree relative had a history of any psychiatric illness, or if there was a second-degree relative with a psychotic disorder. History of psychiatric illness in family members of TDC was assessed with a Dutch translation of the Family Interview for Genetic Studies [20].

Follow-up assessments were conducted on average six years post-baseline and four years after the previous clinical follow-up [17] to determine whether a psychotic transition had occurred. A psychotic syndrome was operationalized as the presence of positive symptoms that were seriously disorganizing, i.e. a score of 6 on any of the items of the SIPS Positive Symptoms subscales for a period of more than 7 days [21]. Additional information on transition to psychosis was obtained by means of a customized semi-structured telephone interview or from medical record. Chart reviews were used to retrospectively confirm psychotic transition by clinical consensus (HvE, PS) and psychotic subjects were subsequently diagnosed according to DSM-IV guidelines (American Psychiatric Association, 1994). TDC subjects were re-assessed for exclusion criteria via clinical interviews and questionnaires.

### Measures

#### Prodromal symptoms and clinical outcome

The SIPS assesses a broad spectrum of prodromal signs and symptoms, categorized in four subscales: positive, negative, disorganization and general symptoms. Symptoms are scored on a 7-point scale from 0 (absent) through 6 (extreme/psychotic intensity). The semi-structured BSABS-P interview assesses subjective disturbances that have shown to be highly predictive of psychosis [19] and are referred to as basic symptoms (BS). The items are scored on a 7-point scale from 0 (absent) through 6 (frequent/extreme) and are summarized in three subscales: cognitive-, perceptual-, and motor disturbances. Each item on the BSABS-P corresponds to a single symptom, which differs in structure from the SIPS in which items are mostly defined by multiple symptoms. In addition, there is evidence suggesting that BS are more prominent in the initial prodromal state and symptoms measured by the SIPS are characteristic of a late prodromal phase, in closer temporal proximity of the onset of psychosis [22].

As a measure of functional outcome, the Global Assessment of Functioning (GAF) scale was used at baseline. The GAF scale is a numeric scale (0 through 100) used by mental health clinicians and physicians to rate social, occupational and psychological functioning. At follow-up, the modified GAF (mGAF) scale was used (0 through 90). It has more detailed criteria and a more structured scoring system than the original GAF. Because of the increased structure, the mGAF scale is more resistant to rater bias [23].

#### Neurocognitive functioning

The test battery consisted of the following measures:

1. General intelligence
  Global intellectual functioning was assessed with the Wechsler Intelligence Scales [24], [25]. Full scale IQ (FSIQ), Verbal IQ (VIQ), and Performance IQ (PIQ) were the dependent variables.
2. Verbal memory
  Verbal memory was assessed using the Dutch 15-Words Task (15WT) [26] that was based on Rey's Auditory Verbal Learning Test [27]. Participants were asked to recall a list of 15 unrelated one-syllable words that was presented repeatedly verbally. Dependent variables were a) total acquisition, i.e. the total score of free recall of five trials (max. 75), and b) retention, i.e. the number of words remembered after 20 minutes delay (max. 15).
3. Psychomotor functioning
  Psychomotor functioning was assessed using a computer-administered finger tapping test (FTT) [28]. Participants were asked to tap their index finger onto a mouse button as often as possible for 10 seconds. The mean number of dominant hand finger tappings over five trials was used in the analyses.
4. Executive functioning (EF)
  The developmental model of EF was adapted from Anderson [29]. This model incorporates four interrelated domains that together enable executive functioning:
*a) Attentional control*
    To measure sustained attention, the no distraction-fast condition of the computer-administered Continuous Performance Test-Identical Pairs version 2.0 (CPT-IP) [30] was administered. Participants were asked to respond as quickly as possible whenever two identical visual stimuli (verbal and nonverbal) were presented in a row. Dependent variable for both conditions was the sensitivity index d'. This measure is computed from the number of hits and false alarms and measures the ability to discriminate a signal from background noise by taking response bias into account.
*b) Working memory and cognitive flexibility*
    Working memory was assessed with a computerized Spatial Working Memory Test (SWMT) [31]. Participants were required to remember the spatial location of a visual stimulus, either immediately after it had disappeared or with a distraction interval of 30 seconds. Dependent variables were the mean distances between target and response in number of pixels for the two conditions separately. The number of perseverative errors on a computerized version of the Wisconsin Card Sorting Test (CST) was used as a measure of cognitive flexibility [32].
*c) Goal setting and problem solving*
    The number of series completed on the CST [32] was used as a measure of problem solving ability and the ability to develop new concepts.
*d) Information processing*
    A verbal fluency (VF) test was used to assess the quality and quantity of verbal output generation. Participants were first asked to name as many words as possible with the initial letter ‘S’ within one minute. Subsequently, they were asked to name words from the semantic category ‘animals’. Dependent variables for this task were the mean numbers of acceptable words produced in each condition.

### Data analysis

Statistical analyses were performed with IBM SPSS version 20.0. Demographic and clinical characteristics were checked for between-group differences (TDC vs UHR and UHR-NP vs UHR-P), using independent samples *t*-test (age), Pearson's *χ2* or, when necessary, Fisher's exact test (gender, inclusion criteria, medication use), and Mann-Whitney tests (parental education, clinical variables). Next, AN(C)OVA was used for between group comparisons of neurocognitive measures. If assumptions for normality of the data and homogeneity of variances were not met, Mann-Whitney tests were used. To reduce the chance of Type I error due to multiple comparisons, but without disproportional inflation of the chance of Type II error, a Dunn-Šidák correction of *p*<0.05 was calculated with the formula 1 – (1 – *α*)^1/*n*^, where *n* is the number of independent neurocognitive tests. Based on seven independent neurocognitive tests, this resulted in a significance threshold of *p*<0.0073. Cohen's *d* was calculated for all variables to estimate effect sizes. In a series of follow-up analyses, binary logistic regression was used to test whether baseline neurocognitive and clinical variables could predict transition to psychosis within the UHR sample. After checking for assumptions and to limit the number of predictors in the model, predictive variables were selected separately for clinical subscales (SIPS and BSABS-P) and neurocognitive variables by using backward stepwise logistic regression (Likelihood ratio method), regardless of significant group differences. To maximize the number of cases in the analysis, the initial neurocognitive model included only those variables that were available for all UHR-P cases. Next, an integrated model focused only on those neurocognitive and clinical variables that were significantly related to transition to psychosis in the previous steps. Cook's distance was used to assess the influence of individual cases, participants with a score >1 were examined and removed from analyses when necessary. Receiver Operator Curves (ROCs) were used to determine sensitivity, specificity and variable cut-off scores. Finally, multiple regression was performed to predict long-term functional outcome for UHR individuals. Only nonredundant predictors that showed a linear relationship with functional outcome were entered into the model. Alpha for all regression analyses was set at 0.05.

## Results

### Group characteristics

Baseline data were available for 67 UHR individuals and 72 TDC (see Table S1 in File S1 for overall group characteristics). Of these individuals, 41 UHR (61%) and 47 TDC (65%) consented to long-term follow-up. Eight out of 41 UHR individuals (19.5%) had experienced a psychotic transition. Two additional UHR individuals without long-term follow-up had experienced psychotic transitions at a previous follow-up [17]. As part of the goal was to predict transition to psychosis, their data from the previous follow-up were included in part of the analyses, resulting in a total of ten UHR-P (23.3%) individuals and 33 UHR-NP individuals. Mean time to transition was 1.3 years (*SD*  = 1.2 y) for UHR-P individuals, with five transitions occurring in the first year post-baseline, another four within the next year and one transition at approximately 4.5 years after inclusion. DSM-IV diagnoses for UHR-P individuals were as follows: 295.30 schizophrenia, paranoid subtype (*n* = 7), 296.04 Bipolar I disorder, psychotic features (*n* = 1), 296.60 schizophrenia, residual type (*n* = 1), 298.9 psychosis - not otherwise specified (*n* = 1). Three TDC (6%) were excluded based on clinical diagnoses received since inclusion (1 epilepsy, 1 posttraumatic stress disorder, 1 affective disorder), resulting in data from 44 TDC for analysis.

There were no significant between group differences for age, gender, parental education or follow-up time. Within the UHR group the UHR-P individuals had slightly higher symptom scores at baseline than the UHR-NP individuals on all clinical variables, which reached significance for SIPS - positive (*U* = 258, *Z* = −2.69, *p* = 0.006) and disorganized symptoms (*U* = 236.5, *Z* = −2.07, *p* = 0.038), as well as BSABS-P - cognitive disturbances (*U* = 212.5, *Z* = −2.59, *p* = 0.008). The UHR-P group also consisted of significantly more individuals who fulfilled the GRD (Fisher's exact, *p* = 0.020) and COGDIS (*χ^2^*_1_ = 5.39, *p*<0.020) criteria at baseline than the UHR-NP group. Forty percent of UHR individuals had used some form of psychotropic medication, but there were no differences in medication use between UHR-P and UHR-NP individuals. At follow-up, global daily functioning was more impaired for UHR-P individuals than for UHR-NP individuals (*U* = 71.5, *Z* = 2.00, *p* = 0.045). Details on demographic and clinical variables are shown in Table 1.

**Table 1:** Group characteristics long-term follow-up sample.

	TDC	UHR	UHR-NP	UHR-P	TDC vs UHR					UHR-NP vs UHR-P		
**Baseline assessment**	(*n* = 44)	(*n* = 43)	(*n* = 33)	(*n* = 10)	*t*/*χ2*/*U*		df	*p*		*t*/*χ2*/*U*	df	*p*
Age in years, M ± SD	15.4±1.3	15.2±2.2	15.0±2.2	15.9±2.4	*t* = 0.55		69		0.587	*t* = −1.07	14	0.302
Gender, N male (%)	23 (52)	27 (63)	19 (58)	8 (80)	*χ2* = 0.98		1		0.321	*χ2* = 1.65	1	0.199
Parental education (y)^a^, M ± SD	13.7±2.1	13.6±1.7	13.7±1.7	13.1±1.7	*U* = 834.5				0.437	*U* = 125		0.314
SIPS/SOPS, M ± SD												
- Positive symptoms	0.4±0.8	8.4±3.9	7.5±3.5	11.4±3.8	*U* = 1854				<0.001	*U* = 258		0.006
- Negative symptoms	0.1±0.3	4.8±4.4	4.2±4.0	6.8±5.0	*U* = 1716				<0.001	*U* = 223.5		0.093
- Disorganized symptoms	0.3±0.6	5.0±3.6	4.3±3.2	7.1±4.1	*U* = 1747.5				<0.001	*U* = 236.5		0.038
- General symptoms	0.4±0.9	6.6±4.3	6.2±4.5	8.2±3.4	*U* = 1776				<0.001	*U* = 216.5		0.141
BSABS-P^b^, M ± SD												
- Cognitive disturbances	0.5±1.0	13.0±8.0	11.0±6.7	19.6±9.0	*U* = 1664				<0.001	*U* = 212.5		0.008
- Perceptual disturbances	0.2±0.5	8.3±7.8	7.5±7.7	11.0±8.1	*U* = 1754				<0.001	*U* = 202		0.105
- Motor disturbances	0.0±0.0	1.5±2.1	1.3±1.7	2.2±3.4	*U* = 1386				<0.001	*U* = 152.5		0.904
GAF, M ± SD	96±6	57±16	58±16	56±15	*U* = 36.5				<0.001	*U* = 150.5		0.681
UHR inclusion criteria^c^												
- APS, N (%)	-	37 (86)	27 (81)	10 (100)	-					*χ2* = 2.1	1	0.146
- BLIPS, N (%)	-	1 (3)	1 (3)	0 (0)	-					*n.a.*		1.000
- GRD, N (%)	-	2 (0)	0 (0)	2 (25)	-					*n.a.*		0.020
- COGDIS, N (%)	-	23 (53)	15 (45)	8 (89)	-					*χ2* = 5.39	1	0.020
Baseline medication^d^, N (%)	-	14 (42)	14 (42)	3 (30)	-					*χ2* = 0.50	1	0.481
- Antipsychotic	-	7 (21)	7 (21)	1 (10)	-					n.a.		1.000
- Antidepressant	-	6 (18)	6 (18)	2 (20)	-					n.a.		1.000
- Psychostimulant	-	1 (3)	1 (3)	0 (0)	-					n.a.		1.000
- Anxiolytic	-	2 (6)	2 (6)	1 (10)	-					n.a.		1.000
- Other	-	1 (3)	1 (3)	0 (0)	-					n.a.		0.495
**Follow-up assessment**												
Age in years^e^, M ± SD	21.3±1.6	21.1±2.4	20.9±2.3	22.2±2.8	*t* = 0.59	68			0.746	*t* = 1.38	39	0.174
Follow-up time in years^e^, M ± SD	5.8±0.7	6.0±1.0	5.9±1.0	6.4±0.9	*t* = 0.76	83			0.450	*t* = 1.33	39	0.190
range	4.8–7.4	3.4–7.9	3.4–7.9	5.3–7.8	*-*					*-*		
Days to transition, M ± SD	-	-	-	488±431	-					-		
range	-	-	-	181–1645	-					-		
mGAF^e^, M ± SD	86±5	58±19	61±18	46±23	*U* = 152.5				<0.001	*U* = 71.5		0.045

a =  Years of education averaged for both parents; ^b^  =  Four BSABS-P scores (3 UHR-NP, 1 UHR-P) were incomplete; ^c^  =  Participants fulfilling multiple criteria were added as a separate individual in each category and for one UHR-P individual the COGDIS criterion could not be evaluated due to missing values; ^d^  =  Participants using more than one type of medication were added as a separate individual in each category; ^e^  =  unavailable for two UHR-P individuals; TDC  =  typically developing controls, UHR  =  Ultra-High Risk; UHR-NP  =  Ultra-High Risk without subsequent psychosis; UHR-P  =  Ultra-High Risk with subsequent psychosis; SIPS/SOPS  =  Structured Interview for the assessment of Prodromal Syndromes/Scale of Prodromal Symptoms; BSABS-P  =  Bonn Scale for the Assessment of Basic Symptoms – Prediction; GAF  =  Global Assessment of Functioning; APS  =  Attenuated Positive Symptoms; BLIPS  =  Brief Limited and Intermittent Psychotic Symptoms; GRD  =  Genetic Risk and a Deterioration in functioning; COGDIS  =  Cognitive Disturbances; mGAF  =  Modified Global Assessment of Functioning.

To check for potential attrition bias, group characteristics were compared between TDC/UHR individuals who participated in the follow-up and those who did not (35 TDC, 24 UHR). TDC with follow-up data were older at baseline (*t*_77_ = −2.16, *p* = 0.034), reported more basic symptoms (*U* = 526, *Z* = −2.62, *p* = 0.009) and had lower GAF scores (*U* = 962, *Z* = 2.03, *p* = 0.043) than TDC who dropped out of the study. There were no such group differences for UHR individuals.

### Baseline comparison of neurocognitive measures

#### TDC vs UHR individuals

Test scores are presented in Table 2. Details of missing data varied per measure and are included in the supplemental information (Table S2 in File S1). TDC had higher scores than UHR individuals on general intelligence measures: FSIQ (*F*_1, 85_ = 8.45, *p* = 0.005, *d* = 0.62) and VIQ (*F*_1, 85_ = 8.98, *p* = 0.004, *d* = 0.64). At a more lenient statistical threshold of *p*<0.05 PIQ (*p* = 0.046) and FTT (*p* = 0.030) also distinguished between groups. On every other neurocognitive task, except for 15WT - delayed recall and SWMT - condition 2, the UHR group performed more poorly than TDC numerically, but these differences did not reach significance. This suggests that, compared to global intelligence measures, more specific neurocognitive skills were relatively spared in the UHR group. Comparisons of the entire baseline sample (67 UHR and 72 controls) on neurocognitive measures produced similar results: all measures of general intelligence significantly differentiated between groups with medium effect sizes (*d*≈0.5; see Table S3 in File S1).

**Table 2:** Baseline cognitive measures for typically developing controls (TDC) and the ultra-high risk groups without (UHR-NP) and with (UHR-P) subsequent psychosis.

	TDC	UHR	UHR-NP	UHR-P	TDC vs UHR				UHR-NP vs UHR-P		
	(*n* = 44)	(*n* = 43)	(*n* = 33)	(*n* = 10)	*F*/*U*	*p*	ES (*d*)		*F*/*U*	*p*	ES (*d*)
**General intelligence**											
FSIQ	109.00±11.04	101.72±12.29	104.30±11.74	93.27±10.61	*F*_ 1,85_ = 8.45	**0.005**	0.62	*F*_ 1,41_ = 6.99		0.012	0.99
VIQ	110.09±11.58	102.70±11.43	104.45±11.70	96.90±8.57	*F*_ 1,85_ = 8.98	**0.004**	0.64	*F*_ 1,41_ = 3.56		0.066	0.74
PIQ	106.16±10.85	100.40±15.37	103.45±15.04	90.30±12.26	*F*_ 1,85_ = 4.10	0.046	0.43	*F*_ 1,41_ = 6.34		0.016	0.96
**Verbal memory**											
15WT direct recall	50.27±9.56	50.05±9.79	50.21±9.48	49.44±11.44	*F*_ 1,84_ = 0.12	0.914	0.02	*F*_ 1,40_ = 0.04		0.838	0.07
15WT delayed recall	10.70±2.74	10.88±2.62	11.00±2.51	10.44±3.12	*F*_ 1,83_ = 0.99	0.820	−0.07	*F*_ 1,40_ = 0.32		0.580	0.20
**Psychomotor functioning**											
FTT dominant hand	58.54±6.25	55.49±6.24	55.41±6.31	55.73±6.38	*F*_ 1,81_ = 4.86	0.030	0.49	*F*_ 1,37_ = 0.19		0.891	−0.05
**Executive functioning**											
CPT-IP numbers - d'	1.15±0.70	0.92±0.69	0.94±0.71	0.87±0.65	*F*_ 1,84_ = 2.20	0.142	0.33	*F*_ 1,40_ = 0.06		0.803	0.10
CPT-IP symbols - d'	1.77±0.69	1.56±0.88	1.48±0.86	1.84±0.91	*F*_ 1,84_ = 1.46	0.230	0.27	*F*_ 1,40_ = 1.20		0.280	−0.41
SWMT condition 1^a^	19.33±8.96	19.76±9.47	18.28±6.90	24.56±14.65	*U* = 887.5	0.386	−0.05	*U* = 163.5		0.262	−0.55
SWMT condition 2^a^	39.00±21.94	38.47±13.91	39.38±15.23	35.56±8.36	*U* = 954	0.132	0.03	*U* = 121.5		0.761	0.31
CST perseverations^a^	6.27±4.21	6.89±3.88	7.24±3.93	5.43±3.51	*U* = 898	0.303	−0.15	*F*_1,34_ = 1.24		0.273	0.49
CST series completed	2.34±1.14	2.11±1.26	2.21±1.24	1.71±1.38	*U* = 719.5	0.471	0.19	*F*_1,34_ = 0.86		0.361	0.38
VF words semantic	22.13±4.91	20.60±5.58	21.03±5.08	19.20±5.08	*F*_1,83_ = 1.86	0.176	0.29	*F*_1,40_ = 0.82		0.371	0.41
VF words letter S	11.19±4.52	10.76±4.83	10.59±5.10	11.30±4.06	*F*_1,83_ = 0.17	0.677	0.09	*F*_1,40_ = 0.16		0.692	−0.15

a Smaller values indicate better performance; TDC  =  typically developing controls, UHR  =  Ultra-High Risk; UHR-NP  =  Ultra-High Risk without subsequent psychosis; UHR-P  =  Ultra-High Risk with subsequent psychosis; FSIQ  =  Full Scale IQ; VIQ  =  Verbal IQ; PIQ  =  Performance IQ; 15WT  =  15 Words Task; FTT  =  Finger Tapping Test; CPT-IP  =  Continuous Performance Test-Identical Pairs; SWMT  =  Spatial Working Memory Test; CST  =  Modified Card Sorting Test; VF  =  Verbal Fluency Test. Significant *p* values are indicated in bold lettertype.

#### UHR-NP vs UHR-P individuals

The data showed that UHR-P had lower FSIQ and PIQ scores than UHR-NP at *p*<0.05, but no group differences remained after correction for multiple comparisons (Table 2). However, effect sizes were large for FSIQ (*d* = 0.99) and PIQ (*d* = 0.96) and medium-to-large for VIQ (*d* = 0.73), suggesting that the lack of significant group differences was a consequence of low statistical power due to small group sizes. For the remaining tasks, the effect sizes were relatively small and not consistently higher or lower for either group.

### Prediction of psychosis

#### Model based on SIPS scales

For SIPS subscales the only significant predictor variable was SIPS positive symptoms (Table 3), suggesting that higher scores on the positive symptoms subscale increased the odds of developing psychosis. The ROC curve indicated that a sensitivity of 40.0% with a specificity of 84.8% was the most optimal classification result, with a cut-off score of 11.5.

**Table 3:** Prediction models of transition to psychosis based on clinical or neurocognitive variables or their combination.

Model 1	B	SE	Wald	*p*	Odds ratio	95% CI	% Sensitivity	% Specificity	% PPV
**SIPS**									
** **Constant	−4.07	0.12	9.81	0.002	0.02				
** **Positive symptoms	0.31	1.30	6.21	0.013	1.36	1.07–1.74	40.0	84.8	44.4
**BSABS-P**									
Constant	−3.62	1.17	9.55	0.002	0.03				
Cognitive disturbances	0.16	0.06	6.06	0.014	1.17	1.03–1.33	66.7	86.7	60.0
**Neurocognition**									
Constant	7.05	3.75	3.54	0.064	1148.18				
FSIQ	−0.08	0.03	4.51	0.034	0.92	0.85–0.99	40.0	97.0	80.0
**Combined**									
Constant	3.02	4.08	3.14	0.459	20.55				
Positive symptoms	0.27	014	3.89	0.049	1.31	1.00–1.72	50.0	90.9	62.5
FSIQ	−0.67	0.04	0.56	0.077	0.93	0.87–1.01			

1 Logistic regression with backward stepwise elimination – final models are displayed (*p* < 0.05); SIPS  =  Structured Interview for the assessment of Prodromal Syndromes; BSABS-P  =  Bonn Scale for the Assessment of Basic Symptoms – Prediction list; FSIQ  =  Full Scale IQ; PPV  =  Positive Predictive Value.

#### Model based on BSABS-P scales

‘Cognitive disturbances’ was the only subscale of the BSABS-P that was a significant predictor, with higher scores associated with increased odds of subsequent psychosis (Table 3). At an optimal cut-off score of 19 the sensitivity was 66.7% and specificity 86.7%. The subscale remained a significant predictor after removal of one influential UHR-P outlier (*p*<0.007).

#### Models based on neurocognitive variables

The initial model included FSIQ, FTT and both VF variables to maximize the number of UHR-P individuals (29 UHR-NP and 10 UHR-P). In the final step, FSIQ was the only variable to remain a significant predictor, with a sensitivity of 40.0% and specificity of 97% (Table 3) and a cut-off score of 86.5. Replacing FSIQ with VIQ or PIQ did not improve the results.

#### Combined clinical and neurocognitive models

**Figure 1:**
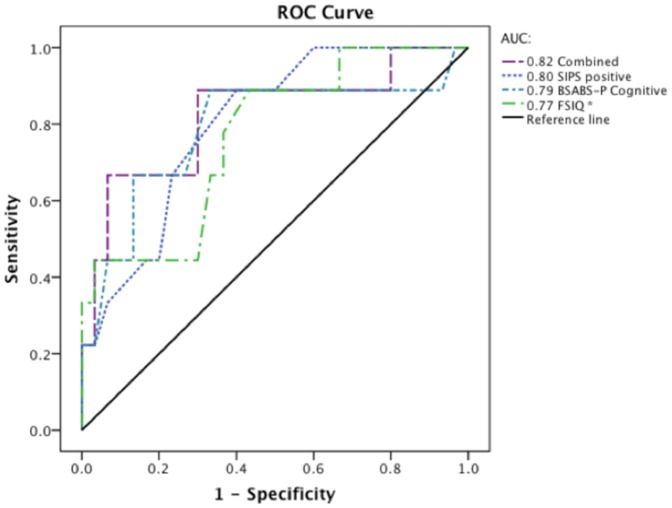
Receiver operating characteristics curves for Structured Interview of Prodromal Syndromes (SIPS) positive symptoms, Bonn Scale for the Assessment of Basic Symptoms – Prediction list (BSABS-P) cognitive disturbances, full-scale IQ (FSIQ) and their combination. * FSIQ scores were transformed to negative values to compare results with the clinical predictor variables.

Two models were tested. First, SIPS positive symptoms and FSIQ were added together to maximize the number of UHR participants (33 UHR-NP and 10 UHR-P). While both predictor variables were retained in the model, only the SIPS ‘positive symptoms’ subscale was significant (Table 3). Next, the BSABS-P ‘cognitive disturbances’ subscale was entered (30 UHR-NP and 9 UHR-P). This variable was discarded after the first step and the remaining model had an overall specificity of 90.9% and a sensitivity of 50.0%. The area under the curve was highest for this model with 6 out of 9 conversions correctly predicted. ROC curves for all predictor variables and their combination are shown in Figure 1. All test variables had satisfactory areas under the curve (±0.8, all *p*<0.05) and the integrated model showed the highest value. In sum, SIPS positive symptoms contributed most to the prediction of psychosis, while adding FSIQ to the model slightly improved classification results.

#### Prediction of functional outcome

**Figure 2:**
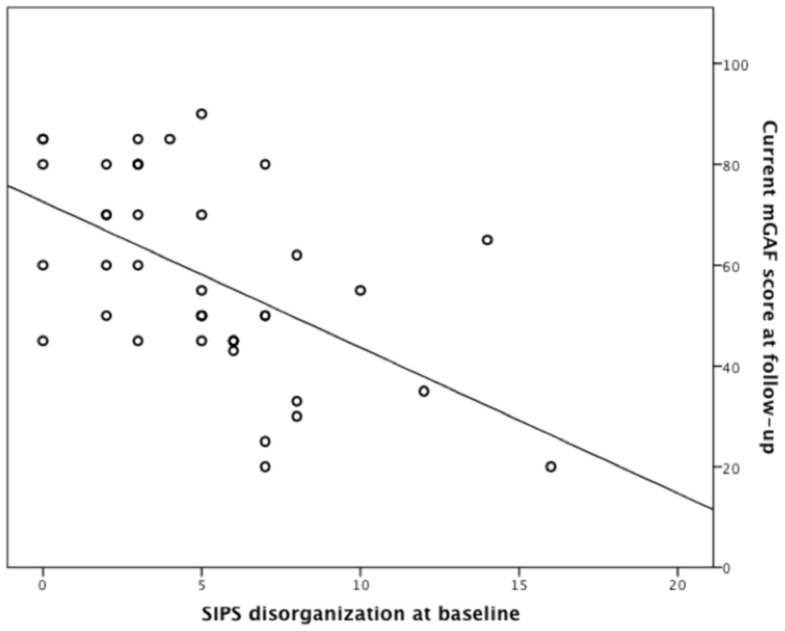
Scatterplot showing a significant linear association between disorganization symptoms (SIPS; X-axis) at baseline and global functioning six years later (mGAF; Y-axis).

Data was available for 41 UHR individuals who completed long-term follow-up. Bivariate correlations were generated to detect linear associations between clinical and neurocognitive variables with mGAF scores at follow-up. The SIPS ‘disorganization’ subscale was the only variable significantly associated with mGAF at follow-up (*r* = −0.55, *p*<0.001). When entered, the resulting model was highly significant (*r^2^* = 0.29, *F*_1,39_ = 16.13, *p*<0.001) and SIPS disorganization was a significant predictor (*β* = −0.54, *t* = −4.02, *p*<0.001; see Figure 2), indicating that a higher score on disorganization symptoms at baseline was predictive of a poorer functional outcome. The regression was repeated with a covariate to check for the influence of time-to-follow-up, but no effect was detected.

## Discussion

The present study investigated whether a combination of neurocognitive parameters and clinical measures at intake could predict clinical outcome at long-term, six-year follow-up in a group of adolescents at UHR for psychosis. There were two main findings: First, we found that UHR individuals had lower IQ scores at baseline than controls and IQ significantly predicted conversion to psychosis, while no other neurocognitive variables discriminated between the groups. Second, both psychotic transition and long-term functional outcome were best predicted by clinical variables and not by neurocognitive measures: Attenuated positive symptoms contributed most to prediction of psychotic transition and global functioning was best predicted by disorganized symptoms. As such, our results suggest that clinical symptoms trump neurocognitive variables in predicting clinical outcome.

### Comparison with previous studies on clinical predictors of psychosis

The added value of this study lies in its combining clinical and neurocognitive variables to predict long-term clinical outcome in adolescents at UHR for developing psychosis. Previous clinical follow-up studies have suggested that attenuated positive symptoms, low functioning and genetic risk combined with functional decline are the most reliable clinical predictors of transition to psychosis [33], [34]. Our study confirms that attenuated positive symptoms at baseline are predictive of psychosis, even at a relatively young age. The criterion of having a genetic risk in combination with functional decline was too rare among our UHR individuals (*n* = 2) to be included as a predictor in this study. Low functioning was not entered into prediction models of psychosis, but did not differ between UHR groups at baseline. In addition to attenuated positive symptoms, the subscale ‘cognitive disturbances’ of the BSABS-P also showed some predictive accuracy for psychosis. Although the small number of UHR-P individuals restricts their interpretation, our results replicate findings from a previous European multicenter study (mean age 23 at baseline) that assessed UHR symptoms with identical clinical instruments [35]. Whereas our results imply that positive symptoms are a more sensitive predictor than cognitive disturbances, the classification outcome, as well as previous findings in larger samples, suggest that they may potentially be used as complementary measures [22].

### Comparison with previous studies on neurocognitive predictors of psychosis

Our neurocognitive findings confirm previously established impairments of general cognition in UHR populations, but are partially at odds with studies reporting impairments in more specific cognitive domains [12], [13], [36], [37]. Similarly, when we exclusively examined neurocognitive variables, psychosis was best predicted by low IQ in this study while previous studies have shown that poorer functioning in more specific (predominantly verbal) neurocognitive domains also have modest predictive capacity (for a recent overview see Lin and colleagues [14]). A number of explanations could account for these discrepancies, such as differences in sample size, neurocognitive measures and follow-up duration. For example, existent relations between cognition and clinical symptoms may have been obstructed by developmental effects, as performance on these types of tasks is highly age-dependent [38] and subclinical symptoms tend to be more frequent and transient in adolescents than in adults [39]–[41]. Although meta-analyses have suggested there may indeed be significant neurocognitive predictors of psychosis [12], [37], results have varied widely across studies and included many negative or potential false positive findings as well [42].

To date only a few studies have considered combining neurocognitive and clinical variables in prediction models for psychosis [13]–[15]. Their outcomes suggest that predictive accuracy of transition to psychosis could be improved by including both neurocognitive and clinical variables. In this study the highest predictive power was achieved by using clinical variables only, although global IQ measures did predict psychosis when entered as a single variable and there was some indication that IQ could contribute to a more optimal group classification when combined with symptom scores. However, a recent North-American multicenter study by Seidman and colleagues [43] also concluded that individual neurocognitive predictors did not improve predictive power beyond clinical models.

### Comparison with previous studies on prediction of functional outcome

A strength of this study is that we did not only focus on transition to psychosis, but also investigated functional outcome as a perhaps more clinically relevant outcome measure of interest. Earlier studies focusing on functional outcome have suggested that negative symptoms and disorganized symptoms may be predictive of functional outcome [44] and that baseline neurocognitive functioning and the course of neurocognitive change in UHR individuals might differentiate between individuals with better or worse functional outcome [6]–[8]. Although the use of domain-specific measures of functional outcome could have potentially been more informative, our results support the general notion that measures of functional outcome are useful assessment tools for long-term clinical prediction studies, as we were able to show that disorganized symptoms are highly predictive of global functioning six years post-baseline. However, we did not find that neurocognitive measures improved prediction of functional outcome as was suggested by the earlier studies. This discrepancy may be due to methodological differences and operationalization of functional outcome. Most previous studies used more domain-specific measures of functional outcome, while the mGAF scale in our study encompasses social, occupational and psychological functioning and thereby has the potential to better characterize global functioning. Similar arguments could provide an explanation for the lack of predictive power for baseline negative symptoms, as well the apparent clinical heterogeneity across and within UHR samples.

### IQ as a vulnerability marker

The finding that low IQ is characteristic of a high-risk profile is consistent with a long history of observations that low premorbid IQ is a risk factor for schizophrenia spectrum disorders [45]. However, UHR studies that investigated the predictive power of IQ have contradictory results. While two studies found that VIQ predicted transition to psychosis [43], [46], most studies have reported that intelligence measures do not predict transition to psychosis [13], [15], [47]. Nevertheless, the hallmark deficit in premorbid global intellectual functioning appears robust from a very young age. Therefore, it is likely that intelligence measures are etiologically relevant, while simultaneously having negligible relevance for individual clinical trajectories. The relative lack of prediction from more specific neurocognitive measures in our adolescent sample suggests that neurocognitive deficits reported in adult UHR individuals may have limited use as early vulnerability markers for psychosis (but see Kelleher and colleagues [48]), in contrast to previously reported structural and functional brain markers [49], [50].

### Methodological considerations

Several limitations of the current study need to be taken in consideration. First, our sample size and the number of UHR individuals who developed psychosis are both relatively small, and therefore the statistical analyses of the prediction of clinical outcome are somewhat underpowered. Ideally, regression analyses include 10 events per predictor variable or more, although smaller numbers can still produce robust results, albeit with a greater risk of introducing bias [51]. Therefore, the results of our regression analyses, in particular for predicting psychotic transition, and ROC curves need to be interpreted with appropriate caution. By correcting for multiple comparisons and restricting the number of predictor variables in our models, we believe we have minimized the chance of reporting on Type I error (false positive findings), with the inevitable drawback of an increased chance of Type II error. Consequently, it is possible that significant contributions of clinical and neuropsychological factors were not picked up in this study. Despite this shortcoming it is also worth noting that longitudinal follow-up studies on young UHR adolescents are rare and a great need has been voiced within the scientific community to validate findings from adult UHR studies in child and adolescent populations [52].

Second, most of the adolescents in our study were help-seeking at an early age [16], while individuals in other UHR cohorts typically do not have a history of contact with mental health services. Accordingly, a relatively high percentage (40%) of our UHR individuals was already using some form of (low-dosage) psychotropic medication at baseline. Arguably, medication may have been prescribed for individuals who were more severely affected clinically, which may in turn have helped prevent the onset of psychosis. However, there were no differences in baseline medication use between those adolescents that went on to develop psychosis and those who did not.

Third, because of the naturalistic design of the study, no systematic data was available concerning non-pharmacological interventions received by UHR participants. Consequently, treatment effects may have further influenced our results. A related limitation is that no standardized instruments were used to assess psychiatric comorbidity in this sample, while findings from a recent study indicate that especially comorbid diagnoses of anxiety and depressive disorders can have substantial impact on later global functioning [53].

In summary, our results suggest that IQ is lower in adolescents at UHR who go on to develop full-blown psychosis, but that its predictive value for transition to psychosis is limited when clinical measures are added to the equation. In this study clinical measures were a more sensitive predictor for both transition to psychosis and long-term functional outcome, in particular attenuated positive symptoms and disorganization. Consequently, these factors are important as vulnerability markers and may be considered a flag for clinical priority in help-seeking UHR adolescents. Furthermore, our results support the idea that it is useful to investigate multiple measures of clinical outcome. Although improving prediction models through long-term longitudinal follow-up is challenging, it is key to improving our understanding of the development of psychosis and associated possibilities for early intervention initiatives.

## Supporting Information

File S1 **Supporting tables.** Table S1, Demographic and clinical characteristics for the total control and UHR samples at baseline. Table S2, Missing data follow-up sample. Table S3, Cognitive performance for the total control and UHR samples at baseline.
(PDF)
