# Supplementary material for: Neurocognitive and Clinical Predictors of Long-Term Outcome in Adolescents at Ultra-High Risk for Psychosis: A 6-Year Follow-Up
Source: PLoS One. 2014 Apr 4;9(4):e93994. doi: 10.1371/journal.pone.0093994 (PMC3976376; doi:10.1371/journal.pone.0093994)
Supplement: File S1 — Supporting tables. Table S1, Demographic and clinical characteristics for the total control and UHR samples at baseline. Table S2, Missing data follow-up sample. Table S3, Cognitive performance for the total control and UHR samples at baseline. (PDF) [file pone.0093994.s001.pdf]

Supplementary information

Table S1. Demographic and clinical characteristics for the total control and UHR samples at baseline

Total baseline sample	TDC (n = 79)	UHR (n = 67)	Statistic	df	*p*
Age in years, M ± SD	15.1 ± 1.5	15.2 ± 2.0	t = - 0.41	1, 131.6	.686
Sex, N male (%)	40 (51)	40 (60)	χ^2 = 1.20	1	.273
Handedness, N right (%)	72 (91)	60 (90)	χ^2 = 0.64	1	.801
Parental education (y), M ± SD	13.8 ± 2.0	13.5 ± 1.8	U = 2028.5		.095
Clinical variables					
- SIPS total, M ± SD	1.7 ± 2.3	24.9 ± 11.5	U = 5212.5		<.001
- BSABS total, M ± SD	1.1 ± 1.5	21.6 ± 14.3	U = 4810.5		<.001
- GAF, M ± SD	88 ± 4	56 ± 14	U = 90.0		<.001
UHR inclusion criteria [a]					
- APS, N (%)		58 (87)			
- BLIPS, N (%)		1 (1)			
- GRD, N (%)		2 (3)			
- COGDIS, N (%)		35 (54)			

[a] = years education averaged for both parents [b] = Participants fulfilling multiple criteria were added as a separate individual in each category; TDC = typically developing controls, UHR = Ultra-High Risk; SIPS = Structured Interview for the assessment of Prodromal Syndromes; BSABS = Bonn Scale for the Assessment of Basic Symptoms; GAF = Global Assessment of Functioning; APS = Attenuated Positive Symptoms; BLIPS = Brief Limited and Intermittent Psychotic Symptoms; GRD = Genetic Risk and a Deterioration in functioning; COGDIS = Cognitive Disturbances

Table S2. Missing data follow-up sample

Task	
FSIQ	-
VIQ	-
PIQ	-
15WT direct recall	1 UHR-P
15WT delayed recall	1 conrol / 1 UHR-P
FTT dominant hand [a]	1 control/ 4 UHR-NP
CPT-IP numbers - d' [b]	1 UHR-P
CPT-IP symbols - d' [b]	1 UHR-P
SWMT condition 1 [b]	2 controls / 4 UHR-NP / 1 UHR-P
SWMT condition 2 [b]	2 controls / 4 UHR-NP / 1 UHR-P
CST perseverations [c]	3 UHR-NP / 3 UHR-P
CST series completed [c]	3 UHR-NP / 3 UHR-P
VF words semantic	1 control / 1 UHR-NP
VF words letter S	1 control / 1 UHR-NP

[a] including two data outliers (UHR-NP); [b] due to technical difficulties; [c] data predominantly missing because UHR individuals were previously assessed with a similar but not identical version of the CST; ; [b] due to technical difficulties; [c] data predominantly missing because of UHR individuals were previously assessed with a similar but not identical version of the CST; FSIQ = Full Scale IQ; VIQ = Verbal IQ; PIQ = Performance IQ; 15WT = 15 Words Task; FTT = Finger Tapping Test; CPT-IP = Continuous Performance Test-Identical Pairs; SWMT = Spatial Working Memory Test; CST = Modified Card Sorting Test; VF = Verbal Fluency Test

Table S3. Cognitive performance in ultra-high risk individuals compared to typically developing controls

	TDC (n = 79)	UHR (n = 67)	F / U	p	ES (d)
FSIQ	107.4 (13.3)	100.0 (12.9)	$F_{1,145}$ = 11.62	< 0.001	0.56
VIQ	108.4 (13.6)	101.9 (12.9)	$F_{1,145}$ = 8.80	0.004	0.49
PIQ	104.7 (12.9)	98.1 (15.1)	$F_{1,145}$ = 8.41	0.004	0.47
15WT direct recall	50.00 (8.50)	49.58 (9.03)	$F_{1,144}$ = 0.09	0.772	0.05
15WT delayed recall	10.81 (2.52)	10.76 (2.60)	$F_{1,143}$ = 0.01	0.674	0.02
FTT dominant hand	57.49 (6.54)	54.91 (6.69)	$F_{1,138}$ = 5.20	0.024	0.39
CPT-IP numbers - d’	1.07 (0.69)	0.87 (0.68)	$F_{1,139}$ = 2.92	0.090	0.29
CPT-IP symbols - d’	1.63 (0.75)	1.55 (0.85)	$F_{1,141}$ = 0.38	0.540	0.10
SWMT condition 1	18.31 (7.23)	20.05 (9.15)	U = 2612.5	0.050	-0.17
SWMT condition 2	39.25 (19.85)	40.37 (15.85)	U = 2584.0	0.129	-0.06
CST perseverations	6.49 (3.93)	7.04 (3.66)	U = 2205.0	0.645	-0.14
CST series completed	2.21 (1.13)	2.11 (1.19)	U = 2054.0	0.804	0.09
VF words semantic	21.69 (4.91)	20.48 (5.08)	$F_{1,141}$ = 2.04	0.155	0.24
VF words letter S	11.44 (4.32)	10.20 (4.64)	$F_{1,141}$ = 2.68	0.104	0.27

TDC = typically developing controls, UHR = Ultra-High Risk; FSQ= Full Scale IQ; VIQ= Verbal IQ; PIQ= Performance IQ; 15WT = 15 Words Task; FTT = Finger Tapping Test; CPT-IP =Continuous Performance Test-Identical Pairs; SWMT = Spatial Working Memory Test; CST = Modified Card Sorting Test; VF = Verbal Fluency Test
